# Near-Infrared Light-Excited Quinolinium-Carbazole Small Molecule as Two-Photon Fluorescence Nucleic Acid Probe

**DOI:** 10.3390/molecules29051080

**Published:** 2024-02-29

**Authors:** Yanqing Sun, Bi Wu, Xinyu Liu, Lixin Liu, Shujing Zhou, Yanru Feng

**Affiliations:** 1 College of Pharmacy, Jiamusi University, Jiamusi 154007, China; 218053084@stu.jmsu.edu.cn (Y.S.); 15765305192@163.com (B.W.); 13199403509@163.com (X.L.); liulixin_2003@163.com (L.L.); zhshj2003@163.com (S.Z.); 2 Heilongjiang Provincial Key Laboratory of New Drug Development and Pharmacotoxicological Evaluation, Jiamusi 154007, China

**Keywords:** two-photon absorption, near-infrared laser, quinolinium-carbazole derivative, nucleic acid DNA probe

## Abstract

This article reports three new two-photon absorption (TPA) materials that are quinolinium-carbazole derivates. They are 3-(N-methyl-4-ethylquinolinium iodide)-9-ethylcarbazole (M4), 3-(N-methyl-4-ethylquinolinium iodide)-9-ethylcarbazole (H2), and 3-(N-methyl-4-ethylquinolinium iodide)-9-ethylcarbazole (H4). Their TPA cross-sections are 491, 515, and 512 GM, respectively. Under the excitation of near-infrared light, their fluorescence emission is about 650 nm. The compounds can stain nucleic acid DNA with the same level of nuclear localization as Hoechst 33342. Under continuous irradiation with a near-infrared laser, the three new compounds showed less fluorescence decay than DAPI, and the average fluorescence decay rates were 0.016%/s, 0.020%/s, and 0.023%/s. They are expected to become new two-photon fluorescent probes of nucleic acid DNA because of their excellent performance.

## 1. Introduction

Nonlinear optical phenomena and effects have attracted increasing attention for a wide range of applications, including optical information technology [1,2], laser technology [3], materials science [4,5], and nano-photonic technology [6,7]. In the mid-1980s, two-photon excitation was applied to the conventional optical microscopy. This technique, called laser scanning confocal microscopy (LSCM) [8,9], was originally developed for application in biology and medicine. Now, it is more and more widely used and has become an essential tool in biomedical experimental research [10,11,12,13]. Compared to conventional fluorescence microscopy, it overcomes the shortcomings of an out-of-focal plane-fluorescence imaging blur and focal plane glare and can improve the optical resolution of fluorescence microscopy. In addition, due to the permeability of near-infrared excitation light, the problems associated with imaging thicker specimens are solved. As a result, it can perform tomography and imaging of samples and provide non-invasive observation and analysis of the three-dimensional structure of cells [14]. With fluorescently labeled probes, the technique can be used to observe fixed cells and tissue sections in dark field cases and to make real-time dynamic observations of the structure, molecules, ions, and life activities of living cells. Therefore, LSCM has become a powerful new research tool in the fields of morphology, molecular cell biology, neuroscience, pharmacology, and genetics [15,16,17,18].

Since 1990, when Dank et al. first used a two-photon laser with a wavelength of 630 nm to obtain the fluorescence images of pig kidney cells with a resolution of 200 nm [19], two-photon fluorescence microscopy imaging has become an important tool for the biomolecular detection and super-resolution tomography of live cells. The emergence of new problems follows with technological advancements. The currently commercially available fluorescence probes, such as the Flou-3, Rhod-1 calcium ion probe, SYTOX green [20], acridine orange DNA, and RNA probe, were developed for the laser-scanning confocal microscope principle of single-photon fluorescence. Because the excitation wavelengths of these probes are mostly in the ultraviolet and visible regions, they are not suitable for LSCM excited by near-infrared (NIR) light. The two-photon fluorescence active-absorption cross-section of these probes is not large enough. It is necessary to increase excitation light intensity to obtain satisfactory fluorescence. In addition, to improve the signal-to-noise ratio and the effect of fluorescence imaging, we also need to increase the laser energy, which often results in a higher fluorescence bleaching effect of the probes [21,22,23]. However, the large laser intensity can easily cause thermal damage to biological samples, thereby interfering with the observation of life phenomena, which largely restricted the application of two-photon technology. Therefore, developing new two-photon fluorescent probes matching with the near-infrared excitation light source is still a challenging task.

In our research, we have designed and synthesized three new quinolinium-carbazole derivatives (H2, M4, and H4). The carbazole ring is linked to the quinoline cation with different substituents through the vinyl group. The measurements of nonlinear optical properties excited by near-infrared light indicate that the synthesized compounds are two-photon absorbing materials. We carried out in vitro titration experiments with DNA and found that the synthesized compounds have the effect of switching the optical properties of DNA. Then, we stained mouse myoblasts (3T3 cells) with synthetic compounds and performed a series of bio-imaging experiments using LSCM, demonstrating that the compounds can serve as two-photon fluorescent probes for the nucleus. Finally, using layered scanning along the z-axis, we successfully achieved 3D imaging of a single 3T3 cell nucleus and measured its size.

## 2. Results and Discussion

### 2.1. Two-Photon Optical Properties

**Figure 1:**
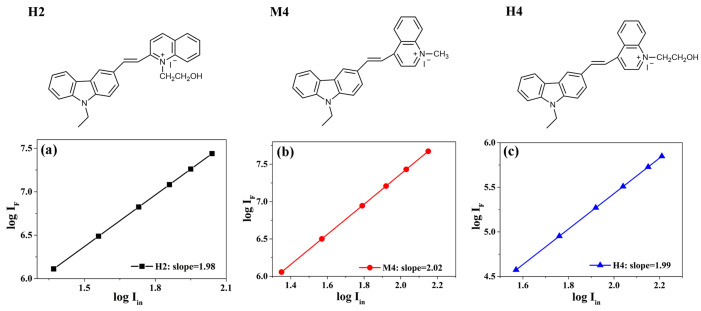
Quadratic log-linear relationship between fluorescence intensity and input laser intensity of H2 (**a**), M4 (**b**), and H4 (**c**) in DMF at 800 nm excitation.

The apparatus measured the TPA cross-section (δ) at different irradiation wavelengths (see Figure S18) with a Ti: a sapphire laser system (80 fs, 80 MHz), using compound M4 as a representative. The results are shown in Figure S19. When the radiation wavelength is between 750 and 830 nm, the δ of M4 first increases and then decreases, and the maximum is at 800 nm. Since the compounds H2, M4, and H4 are similar in structures, the TPA properties of the new compounds were measured in DMF using a femtosecond laser pump source at 800 nm. Then, we measured the fluorescence-emission spectra of the compounds H2, M4, and H4 at various powers (i.e., the excitation light energy) and calculated the logarithm of the excitation light power and the respective fluorescence intensity. This was performed to determine the dependence of the two-photon-induced fluorescence-excitation intensity of the compound on the incident-light intensity, and the results are shown in Figure 1. The slopes of the lines are all close to 2, indicating that the compounds H2, M4, and H4 are two-photon-absorbing (TPA) materials.

Next, we measured the one-photon excitation fluorescence (OPEF) and two-photon excitation fluorescence (TPEF) emission spectra of the compounds. Then, we calculated the fluorescence quantum yield (*φ*) and the two-photon-absorption cross-section (*δ*) of the compound as [24,25].

(1) $$\varphi =\varphi_{s}\left(\frac{A_{s}\left(\lambda_{s}\right)}{A\left(\lambda \right)}\right)\left(\frac{n^{2}}{{n_{s}}^{2}}\right)\frac{\int F}{\int F_{s}}$$

(2) $$\delta =\delta_{s}\frac{\varphi_{s}C_{s}}{\varphi C}\frac{F}{F_{s}}$$

**Figure 2:**
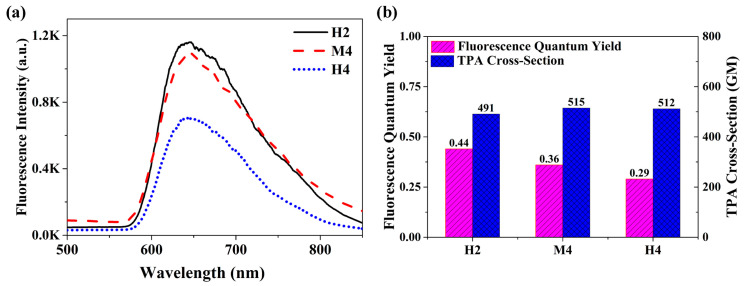
(**a**) Two-photon fluorescence spectra of H2, M4, and H4 in DMF at an excited wavelength of 800 nm laser in DMF, and (**b**) fluorescence quantum yield and two-photon-absorption cross-sections of H2, M4, and H4 in DMF.

Here, *s* represents the reference chemical, *A* and *F* refer to the absorption intensity and fluorescence intensity, respectively, and *C* and *n* refer to the concentration and refractive index of the examined solution, respectively. The ARhodamine 6 G ethanol solution (*φ*_s_ = 0.95, *δ*_s_ = 210 GM) was selected as the reference [26]. The TPEF spectra of compounds H2, M4, and H4 are shown in Figure 2a, and the *φ* and *δ* results are shown in Figure 2b.

These new compounds’ fluorescence emission ranges from 600 to 800 nm, and their maximum emission wavelength is about 650 nm, which effectively avoids the endogenous fluorescence interference from the biological object itself. The TPA cross-sections of compounds H2, M4, and H4 induced by the 800 nm femtosecond pumped laser are 491, 515, and 512 GM, and the fluorescence quantum yields are 0.44, 0.36, and 0.29, which are much larger than that of the commercial nucleic acid two-photon probe, DAPI (only 0.46 GM) [27]. In fact, the TPA cross-section values are similar to the two-photon nucleic acid probes of pyridinium-carbazole reported recently, which have structures similar to these new compounds [28,29,30,31]. Despite the different solvents, the two-photon action cross-sections (*φ* × *δ*) of the three new compounds H2, M4, and H4 were obviously larger than those reported. It shows that the luminescence of new compounds is much higher in DMF. This is due to the modification of the structures by the quinoline cation, which increases the conjugated system and is beneficial to the delocalization of electrons, resulting in an increase in the TPA cross-section of the compounds. These two-photon properties make it possible for the new compounds to function as two-photon fluorescent probes.

### 2.2. DNA Optical On–Off Effect

To explore whether these new compounds have the effect of switching the optical properties of DNA, we conducted a series of in vitro titration experiments using calf thymus DNA (ct-DNA) and determined their linear absorption and emission spectra and two-photon emission spectra. The results are shown in Figures S10–S17. In the absorption spectra, we found that as ct-DNA concentration increased, the absorption peaks of the compounds all showed a weak redshift, and the absorption intensity decreased first and then gradually increased (Figures S10–S12). The difference is that there is a significant increase in both the OPEF and TPEF emission spectra. As the concentration of DNA increased from zero to saturation, the fluorescence intensity increased about 2- to 4-fold (Figures S14–S16 and S20–S22), and an optical on–off effect of DNA was observed. In comparison with a blank test of DNA titration (Figures S13 and S17), it was found that the changes in the fluorescence emission behavior were caused by the interaction between the compounds (H2, M4, and H4) and DNA rather than the solvent and DNA.

### 2.3. Bio-Imaging of Colocalization Experiment

**Figure 3:**
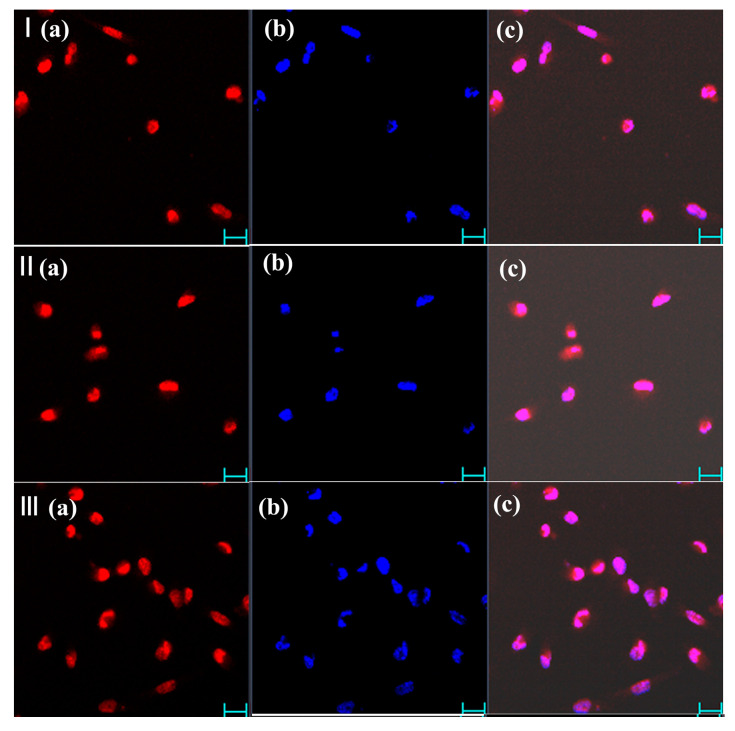
Confocal fluorescent images of 3T3 cells double-stained with H2 (**Ia**, 0.3 μM), M4 (**IIa**, 0.3 μM), H4 (**IIIa**, 0.3 μM), and Hoechst 33342 (**b**, 0.3 μM) for 15 min. (**a**) Images of H2 (**I**), M4 (**II**), and H4 (**III**), λex = 405 nm and λem = 580–650 nm. (**b**) Images of Hoechst 33342, λex = 330–380 nm and λem = 410–490 nm. (**c**) Overlay images of (**a**,**b**). Scale bar = 20 μm.

To prove the potential application value of compounds H2, M4, and H4 as nucleic acid probes, we performed a series of double-stained experiments of 3T3 cells using these compounds and a commercial DNA fluorescent marker Hoechst 33342, respectively. The results are shown in Figure 3.

The results shown in Figure 3(Ia,c–IIIa,c) have a high degree of reproducibility, indicating that compounds H2, M4, and H4 can penetrate the cell membrane and nuclear membrane, enter the nucleus, and selectively stain DNA. Upon light induction, fluorescence is emitted, and the nuclei are imaged; thus, the synthesized compounds have the same nuclear localization ability as Hoechst 33342. By calculating the number of limited nuclei in Figure 3(Ia,c–IIIa,c), the colocalization coefficients were found to be 0.91, 0.90, and 0.90, respectively. We know that Hoechst 33342 is a mature marker for commercial nucleic acid staining. Since the synthetic compounds have a highly consistent nucleic acid localization level, the application of these novel compounds as nucleic acid probes can be explored.

### 2.4. One- and Two-Photon Bio-Imaging

**Figure 4:**
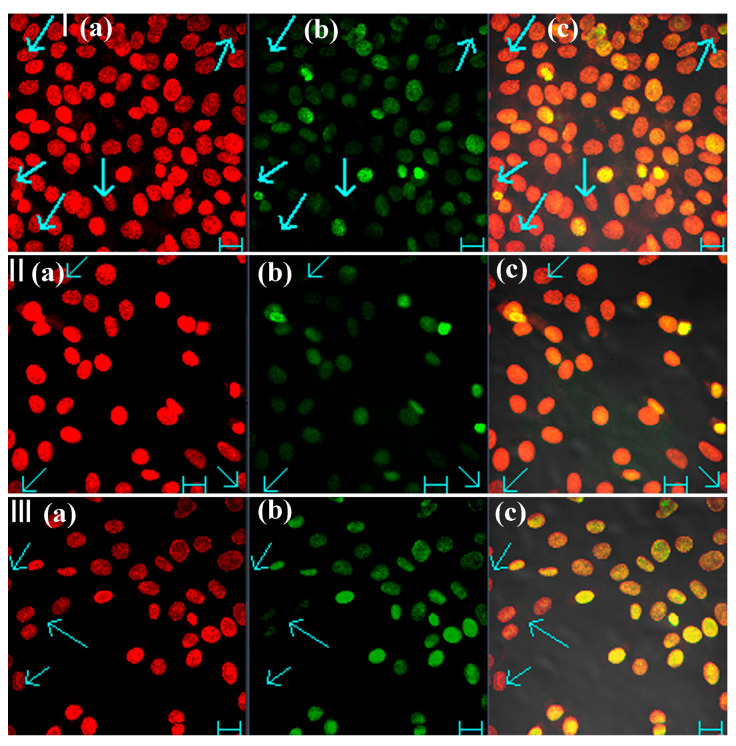
One- and two-photon confocal fluorescence images of 3T3 cell nuclei stained with H2 (**I**, 0.3 μM), M4 (**II**, 0.3 μM), and H4 (**III**, 0.3 μM) for 15 min. (**a**) TPEF biological images, λex = 800 nm and λem = 580–650 nm. (**b**) OPEF biological images, λex = 400 nm and λem = 580−650 nm. (**c**) Overlay images of (**a**,**b**). Scale bar = 20 μm.

To determine whether compounds H2, M4, and H4 are more suitable as two-photon fluorescence probes for nucleic acids, we carried out one- and two-photon imaging contrast testing of compound-stained 3T3 cells. The results are shown in Figure 4.

We found that a greater number of 3T3 nuclei can be clearly imaged by 800 nm light (Figure 4a) than by 400 nm light (Figure 4c). The nuclei indicated by the arrows in the images can be clearly seen in Figure 4a but are hardly visible in Figure 4c. This demonstrates that two-photon fluorescence imaging is superior to one-photon imaging. This difference in imaging quality is attributed to Rayleigh scattering. The longer the wavelength of the incident light, the smaller the scattering intensity; conversely, the greater the scattered light intensity. The greater the scattered light intensity, the stronger the background fluorescence and the greater the signal noise, which is obviously not conducive to clear imaging. Therefore, fewer nuclei were clearly visualized by one-photon fluorescence imaging compared to two-photon imaging in the experiment. When LCSM was employed to perform the procedure, because the intensity at the focal point is much greater than the non-focused regions, Rayleigh scattering had the greatest effect on imaging the nuclei at the edge. Particularly about the nuclei at the edges in one-photon fluorescence imaging, Rayleigh scattering has the greatest impact. Two-photon excitation has a longer excitation wavelength, and the intensity of Rayleigh scattered light is less than that of single-photon fluorescence so that the edge nuclei can be imaged more clearly. This advantage has practical application value in medical diagnosis and testing. 

### 2.5. Photo-Stability Experiment

**Figure 5:**
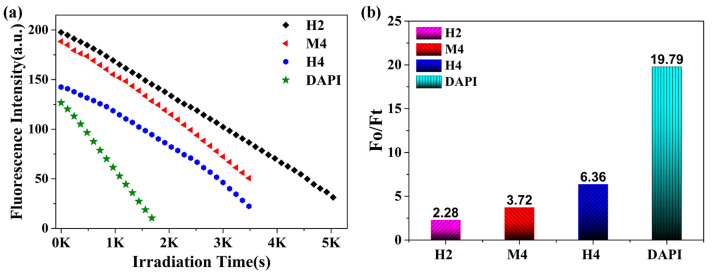
Photo-stability comparison of H2, M4, H4, and DAPI, (**a**) fluorescence decay as a function of time under successive irradiation (800 nm); (**b**) multiples of fluorescent attenuation during 0–30 min.

When laser-induced fluorescence is used for imaging, it is necessary to increase the energy of the excitation light to improve the signal-to-noise ratio and sensitivity, thereby enhancing the imaging quality. However, fluorescent probes, which are bio-object markers, can generate fluorescent bleaching and even photo-toxicity when irradiated by high-energy lasers. Therefore, for a fluorescent probe to achieve excellent performance, it must have high light stability. To explore the photo-stability of the newly synthesized compounds, we captured nuclear fluorescence images of the stained 3T3 cells at intervals of 60 s under continuous laser irradiation (see Figures S23–S26) and recorded the fluorescence intensity at fixed points. The dependence of the fluorescence intensity on time was found and compared with that of the commercially available nucleic acid two-photon fluorescent probe, DAPI (Figure 5), to demonstrate the performance of the compounds in terms of light stability.

As seen in Figure 5a, the fluorescence decay rates of compounds H2, M4, and H4 were much lower than that of DAPI, which were 0.016%/s, 0.020%/s, 0.023%/s, and 0.026%/s (DAPI), respectively. We calculated the total fluorescence decay multiples after 30 min (1800 s), and those of H2, M4, H4, and DAPI were 2.28, 3.72, 6.36, and 19.79-fold (Figure 5b), respectively. The photo-stability of these new compounds was found to be significantly better than that of DAPI. Therefore, they are expected to be better two-photon fluorescence probes.

In addition, we also noticed that the fluorescence intensity decay multiples and average decay rates of compound H2 are lower than M4 and H4, indicating that the photo-stability of 2-methyl quinolinium cation-substituted carbazole derivatives is higher than that of 4-methyl quinolinium. The fluorescence attenuation may be attributed to DNA injury due to the sustained femtosecond laser excitation, which gradually weakened until the binding force between the compound (including DAPI) and the DNA molecular was eliminated. When the complex of DNA with a fluorescent probe is excited by a continuous high-power laser, a large amount of thermal energy is retained due to the slowness of energy dissipation in the presence of base pairs. This could cause DNA molecules to unwind the helical or break-strand structure [32]. Then, the dissociation of the DNA-probe complexes caused a decrease in fluorescence intensity till it disappeared. Thus, long-sustained femtosecond laser excitation can cause DNA thermal damage and lead to fluorescence decay. Suppose the TPA activity cross-section of the fluorescent probe is larger. In that case, most of the incident laser energy is absorbed by the probe and converted into light energy of fluorescence emission, and the remaining small amount of excitation light energy into thermal energy. Therefore, a larger two-photon-absorption active cross-section is beneficial to reduce the DNA thermal damage rate and slow down the destruction of the DNA-probe complex. Compounds with large two-photon active cross-sections act as probes for DNA that can withstand longer irradiation times and provide higher fluorescence intensity for near-infrared excitation [24]. In fact, the TPA action cross-section of the three new compounds is much larger than DAPI. As a result, under continuous laser irradiation, the fluorescence bleaching is much smaller than DAPI. Meanwhile, for the three new compounds, the two-photon action absorption cross-section of H2 is the largest, M4 is the second, and H4 is the smallest. Therefore, the weakest fluorescent bleaching effect is H2, followed by M4, and H4 has the most obvious fluorescence attenuation among them.

### 2.6. Cell 3D Bio-Imaging

**Figure 6:**
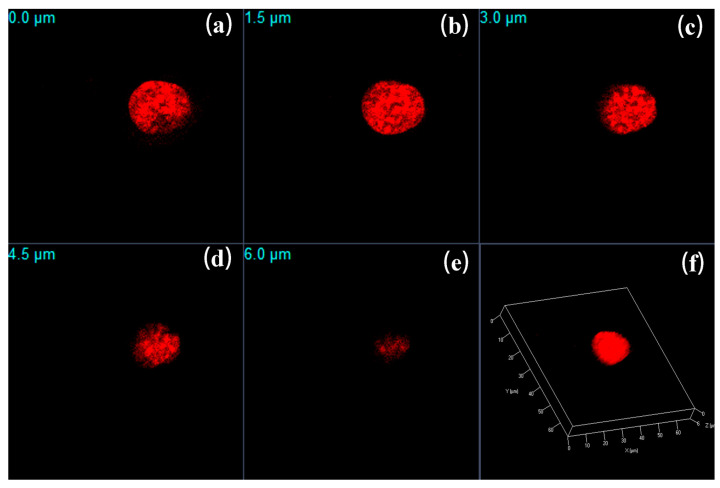
Two-photon confocal fluorescent images of 3T3 cells stained with M4 (0.3 μM) for 15 min for various penetration depths in the 0–6 μm range. The wavelength for the two-photon excitation was 800 nm. Scale bar = 20 μm. Magnification: 63× ((**a**–**e**) nucleus fluorescent images at various depths, (**f**) nucleus 3D fluorescent image).

Near-infrared light is less affected by scattering than ultraviolet–visible light; it easily penetrates samples and has less photo-toxicity. Therefore, near-infrared light is more suitable for observing thick samples and living cells. We used a Leica DMI 3000B inverted microscope and a Carl Zeiss LSM710 laser-scanning confocal microscope to observe the fluorescence imaging results, and we recorded images of M4-stained 3T3 cells at various optical axis depths. The images were processed by Zeiss Zen Lite 2012 software, and the results are shown in Figure 6. We took fluorescence images of the 3T3 nuclei every 1.5 μm along the optical axis. Figure 6b,c show that the nucleus had the highest fluorescence intensity at 1.5 μm and 3.0 μm, and the cell nucleus was completely oval; the fluorescence intensity at 0 μm and 4.5 μm was slightly weaker, and the structural integrity of the cell nucleus was also lower; the fluorescence intensity at 6.0 μm was the weakest. Measurement showed that the diameter of the cell nucleus was 6 μm. Employing LSCM and matched two-photon fluorescence probes can help us visualize the changes in nuclei under various circumstances and better understand clear cell nuclei under microscopic and dark field conditions. This is of great significance in the biomedical field. In addition, it is worth emphasizing that cell nuclei are complex structures. Inspection of the stained nuclei in this study indicates that these three new dyes stain nuclear chromatic but not the nucleoli. In keeping with this, there is no significant staining of potentially nucleic acid [dsRNA] ]-rich cytoplasm. It is a little surprising that these cations are nucleic acid stains and do not give rise to any staining of mitochondria since the dyes are probably somewhat lipophilic.

## 3. Experimental Section

### 3.1. Materials and Chemicals

Chemical reagents, including iodomethane, 2-iodoethanol, *N*-ethylcarbazole, *o*-ethylquinoline, and *p*-methylquinoline, were purchased from J & K Chemicals (Beijing, China). There were also some other chemical reagents purchased from Sinopharm Chemical Reagent Co., Ltd. (Shanghai, China), such as piperidine, phosphorus oxychloride (POCl_3_), dimethyl formamide (DMF), acetone, and so on. All reagents used in the chemical reactions were of analytical grade. Biological reagents, including ct-DNA, were purchased from Biodee Biotechnology Co., Ltd. (Beijing, China), DAPI was purchased from Sigma Co., (St. Louis, MO, USA) and Hoechst 33342 was purchased from Invitrogen Co. The 3T3 Cell was purchased from Shanghai Biya Biotechnology Co., Ltd., (Shanghai, China) SMMC-7721 Cells were purchased from Shanghai Biya Biotechnology Co., Ltd. Dulbecco’s Modified Eagle Medium (DMEM) was purchased from Shanghai Biological Technology Development Co., Ltd., (Shanghai, China). The Tris-HCl-NaCl buffer solution (10 mmol Tris, 100 mmol NaCl, pH = 7.2) was formulated and stored at 4 °C before use. The concentration of ct-DNA in the buffer solutions was determined to be approximately 1.5 × 10^−3^ M.

### 3.2. Synthesis

**Scheme 1:**
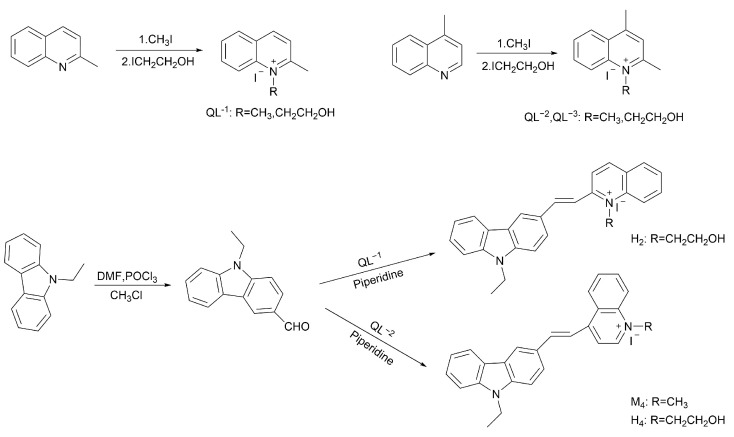
Synthesis of the quinolinium-carbazole derivatives.

The target compound was generated by the Vilsmeier–Haack reaction and the aldol condensation reaction using N-ethylcarbazole and quinolinium as the raw material. The synthesis route is shown in Scheme 1.

*N*-ethyl-3-formyl carbazole was synthesized through the Vilsmeier–Haack reaction from *N*-ethyl-carbazole, as described in the literature [33,34]. *N*-ethoxyl-2-methyl-quinolinium iodide (QL^−1^), *N*-methyl-4-methyl-quinolinium iodide (QL^−2^), and *N*-ethoxyl-4-methyl-quinolinium iodide (QL^−3^) were synthesized through a salinization reaction from 2-methylquinoline, 4-methylquinoline, 2-iodoethanol, and iodomethane as described in [28].

The synthesis of 3-(*N*-methyl-4-ethylquinolinium iodide)-9-ethylcarbazole (M4), *N*-ethyl-3-formyl carbazole (2 mmol), and QL^−2^ (1.1 mmol) was dissolved in 20 mL of methanol, and three drops of piperidine were added under stirring. The solution was heated to 80 °C and refluxed for 2 h, and then cooled to room temperature. After filtration, the product was washed several times with methanol. Recrystallized by methanol, a red powder was obtained with a yield of 82.9%.

The 3-(*N*-methyl-4-ethylquinolinium iodide)-9-ethylcarbazole (H2) and 3-(*N*-methyl -4-ethylquinolinium iodide)-9-ethylcarbazole (H4) were synthesized from *N*-ethyl-3-formyl carbazole and QL^−1^ or QL^−3^ by the same approach. The characterization data of the synthesized compounds and MALDI-TOF-MS, ^1^H-NMR, and ^13^C-NMR are shown in the Supplementary Information (Figures S1–S9).

### 3.3. Spectroscopic Measurements

The normalized optic properties of synthesized compounds M4, H2, and H4 were determined by analysis using a UV/VIS/NIR 2250 spectrophotometer and a 970CRT fluorescence spectrometer (Agilent Technologies Co., Ltd., Santa Clara, CA, USA). Their nonlinear optical properties were determined under the following conditions: Ti: A sapphire laser system was applied with a pulse duration of 80 fs and a repetition rate of 80 MHz, as well as a 7ISW301 spectrometer equipped with a 7IDA1 data-acquisition unit and a 7IP1100 high voltage-regulated power (DEC Precision Technology Co., Ltd., (Shenzhen, China)). All optical behaviors were measured in a DMF solution at a compound concentration of ~10^−5^ M.

### 3.4. Cell Culture and Bio-Imaging Experiment

It is well known that deoxyribonucleic acid (DNA) is mainly distributed in the nucleus, and smaller amounts are present in the chloroplast and mitochondria. We investigated whether the synthetic compound successfully stains the nucleus and allows efficient imaging through a series of bio-imaging experiments. A Leica DMI 3000B (Leica Microsystems Trading Co., Ltd., (Shanghai, China)) inverted microscope and A Carl Zeiss LSM 710 (Leica Microsystems Trading Co., Ltd., (Shanghai, China)) confocal scanning microscope were used for the series of biological fluorescence imaging trials. Mouse myoblasts 3T3 cells (ATCC No. CCL-163.2, Biya Biotechnology Co., Ltd., Shanghai, China) were used for the cell culture; they were grown on coverslips of Petri dishes at 25 °C in a culture solution (Dulbecco’s Modified Eagle’s Medium DMEM) under 5% CO_2_ for 12 h. After washing and fixation, the 3T3 cells were stained with H2, M4, H4, Hoechst 33342, and DAPI in a process conducted at 37 °C for 15 min. The concentration of the fluorescent stain was 0.3 μM.

## 4. Conclusions

In conclusion, these three newly synthesized compounds are typical two-photon-absorbing materials. Under irradiation with near-infrared light, these compounds emit up-converted fluorescence around 650 nm. Their two-photon absorption cross-section exceeds 140 GM, which is about 4–10 folds that of the pyridinium-carbazole derivative two-photon fluorescence probe. These new compounds can penetrate cell membranes and stain nuclear chromatic, but not the nucleoli. The experimental results show that they have the same level of nuclear localization as Hoechst 33342. Thus, the three new compounds can be used as new fluorescent nucleic acid markers. These new compounds have good photo-stability and low fluorescence attenuation. With the aid of LCSM’s layered scanning technology, three-dimensional imaging of the nucleus can be achieved. We believe that this research will lead to the realization of new types of two-photon fluorescent probes that can achieve better performance in comparison to previous probes.

## Data Availability

Data are contained within the article and Supplementary Materials.

## Supplementary Materials

The following supporting information can be downloaded at: https://www.mdpi.com/article/10.3390/molecules29051080/s1, Figures S1–S9: The characterization data of the synthesized compounds (H2, M4 and H4) and MALDI-TOF-MS, ^1^H-NMR, and ^13^C-NMR; Figures S10–S17: Linear absorption and emission spectra and two-photon emission spectra of compounds (H2, M4 and H4); Figure S18: Experiment apparatus schematic diagram for two-photon-excited properties; Figure S19: TPA cross-sections of M4 under different incident wavelength; Figures S20–S22: Two-photon excited fluorescence spectrum of H2, M4 and H4 according [ctDNA] increased; Figures S23–S26: Time-dependent two-photon confocal fluorescence images of 3T3 cell nucleus stained with H2, M4, H4 and MDPI.
