# Supplementary material for: Near-Infrared Light-Excited Quinolinium-Carbazole Small Molecule as Two-Photon Fluorescence Nucleic Acid Probe
Source: Molecules. 2024 Feb 29;29(5):1080. doi: 10.3390/molecules29051080 (PMC10934762; doi:10.3390/molecules29051080)
Supplement: Supplementary file 1 [file molecules-29-01080-s001.zip › molecules-2863739-supplementary.pdf]

Supplementary information

Near-infrared light excited quinolinium-carbazole small molecule as two-photon fluorescence nucleic acid probe

Yanqing Sun [1], Bi Wu [1], Xinyu Liu [1], Lixin Liu [1,2], Shujing Zhou [1,2] and Yanru Feng [1,2,*]

1 College of Pharmacy, Jiamusi University, Jiamusi, 154007, China

2 Heilongjiang Provincial Key Laboratory of New Drug Development and Pharmacotoxicological Evaluation, Jiamusi, 154007, China

*Corresponding author: nancy_fyr@163.com

Table of Contents

1. MALDI-TOF-MS spectra, ^{1}H-NMR, and ^{13}C-NMR of the compounds **(Figure S 1–9).**
2. Electronic absorption spectrum and normalized fluorescence spectrum of **H2, M4, H4**, and blank Tris-HCl buffer solution with DNA titration **(Figure S 10–17).**
3. Two-photon excited titration fluorescence spectra of **H2, M4,** and **H4 (Figure S 18–22).**
4. Time-dependent two-photon confocal fluorescence images of 3T3 cell nucleus stained with **H2**, **M4**, **H4**, and **DAPI (Figure S 23–26)**

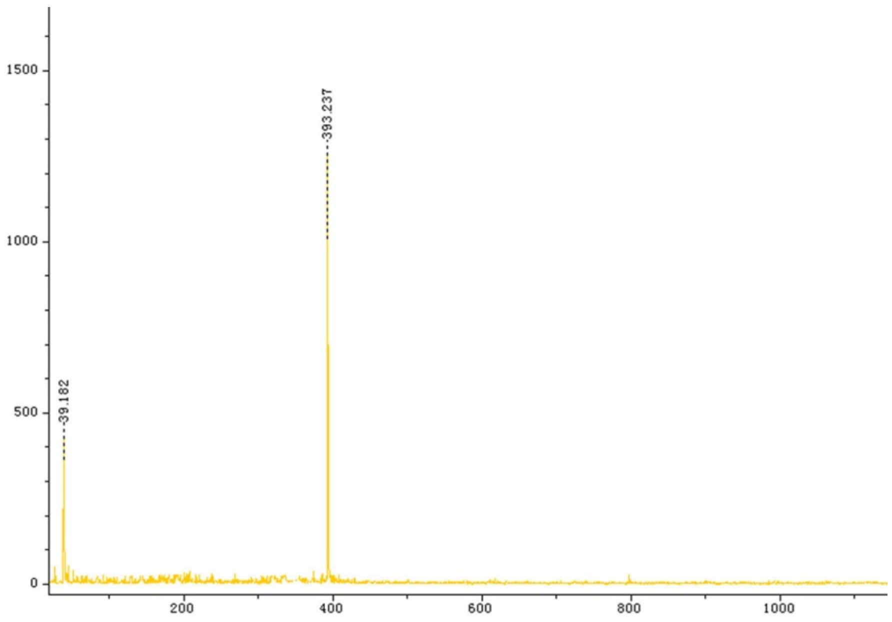

Figure S1 MALDI-TOF-MS spectra of H2

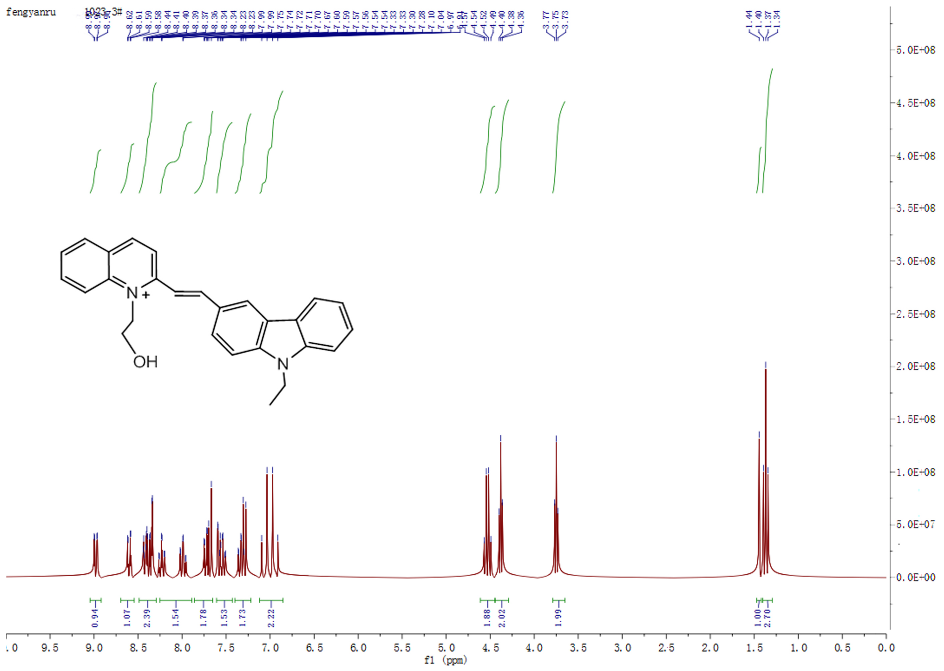

Figure S2 ^{1}H-NMR of H2 (400 MHz, DMSO-d6)

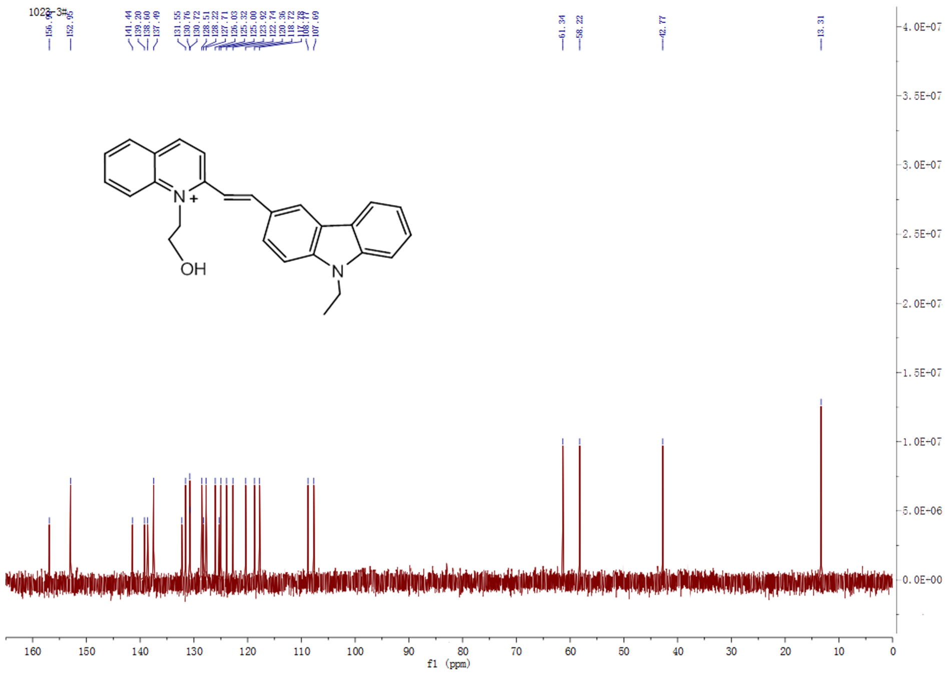

Figure S3 ^{13}C-NMR of H2 (125 MHz, DMSO-d6)

H2:

Mp: 260.6~260.9 °C; ^{1}H-NMR (400 MHz, DMSO-*d6*) δ (ppm): 7.04~9.05 (m, 13H), 6.97 (d, 1H), 6.91 (d, 1H), 4.52 (q, 2H), 4.38 (t, 2H), 3.75 (t, 2H), 1.44 (s, 1H), 1.37 (t, 3H). ^{13}C-NMR (125 MHz, DMSO-*d6*) δ (ppm): 156.90, 152.95, 141.44, 139.19, 138.60, 137.49, 132.24, 131.55, 130.76, 130.73, 128.51, 128.22, 127.71, 126.03, 125.32, 125.00, 123.92, 122.74, 120.36, 118.72, 117.78, 108.77, 107.69, 61.34, 58.22, 42.77, 13.31. MALDI-TOF-MS: m/z calculated for $[C_{27}H_{25}N_2O]^+$, 393.50; experiment: 393.237.

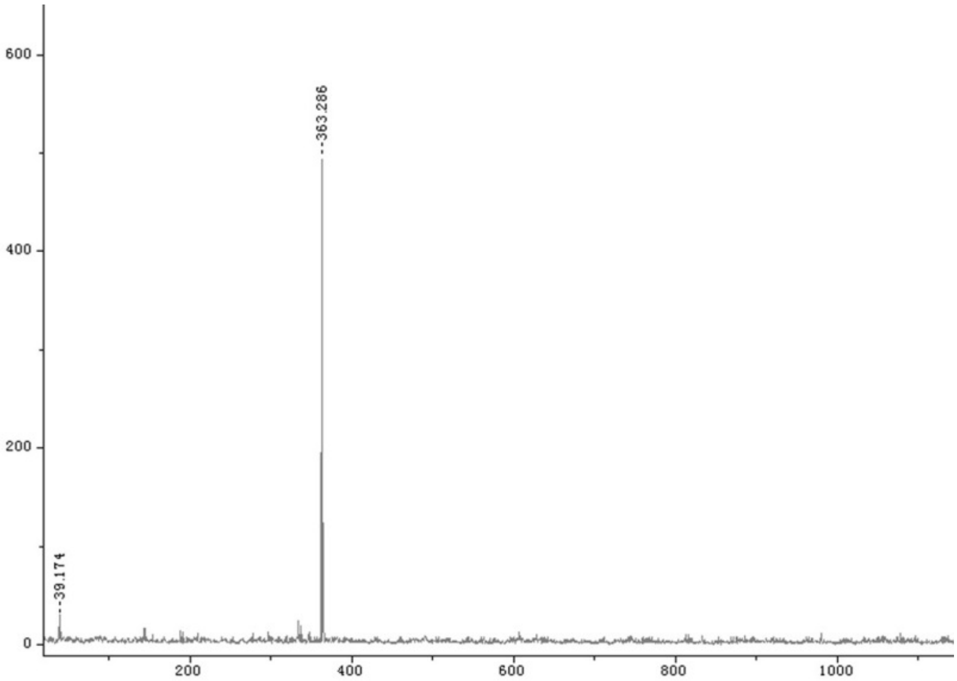

Figure S4 MALDI-TOF-MS spectra of M4

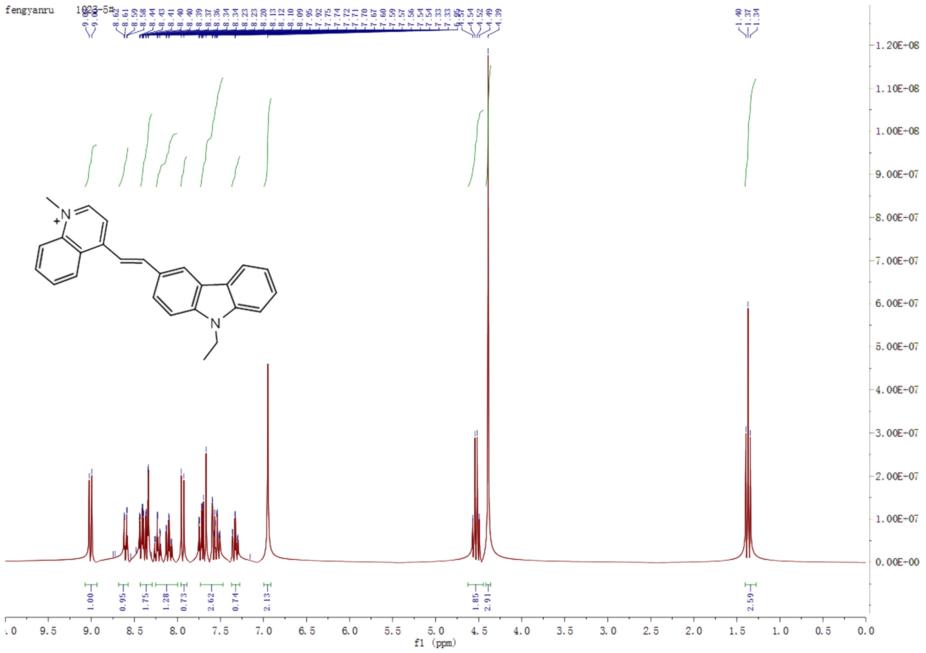

Figure S5 ^{1}H-NMR of M4 (400 MHz, DMSO-d6)

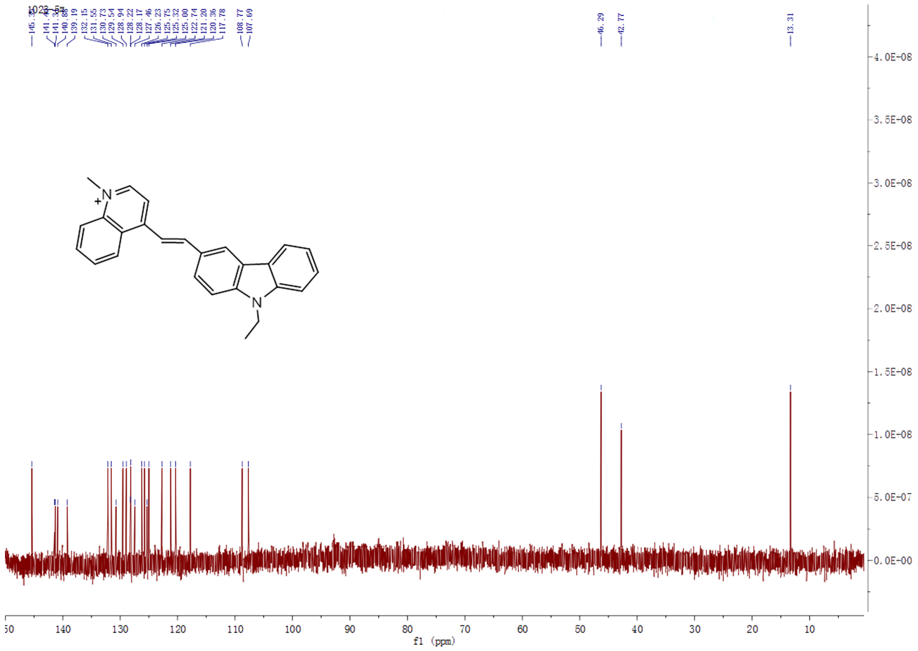

Figure S6 ^{13}C-NMR of M4 (125 MHz, DMSO-d6)

M4:

Mp: 268.3~269.0 °C; ^{1}H-NMR (400 MHz, DMSO-*d6*) δ (ppm): 7.33~9.03 (m, 13H), 6.95 (s, 2H), 4.52 (q, 2H), 4.39 (s, 3H), 1.37 (t, 3H). ^{13}C-NMR (125 MHz, DMSO-*d6*) δ (ppm): 145.35, 141.44, 141.31, 140.88, 139.19, 132.15, 131.55, 130.73, 129.54, 128.94, 128.22, 128.17, 127.46, 126.23, 125.75, 125.32, 125.00, 122.74, 121.20, 120.36, 117.78, 108.77, 107.69, 46.29, 42.77, 13.31. MALDI-TOF-MS: m/z calculated for $[C_{26}H_{23}N_2]^+$, 363.47; experiment: 363.286.

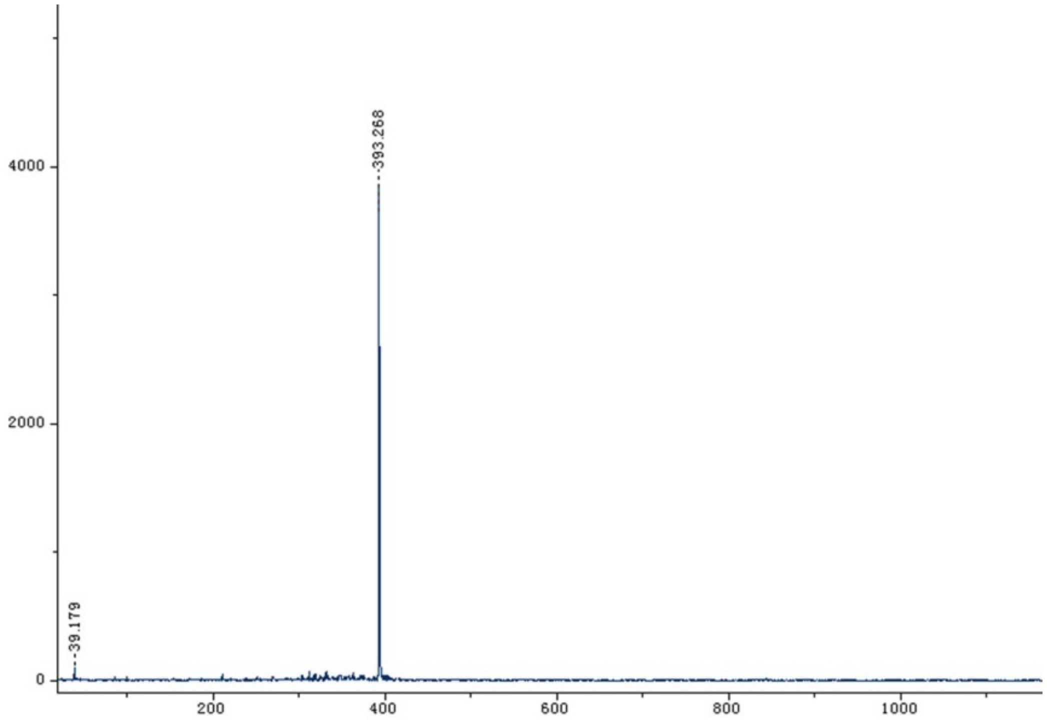

Figure S7 MALDI-TOF-MS spectra of H4

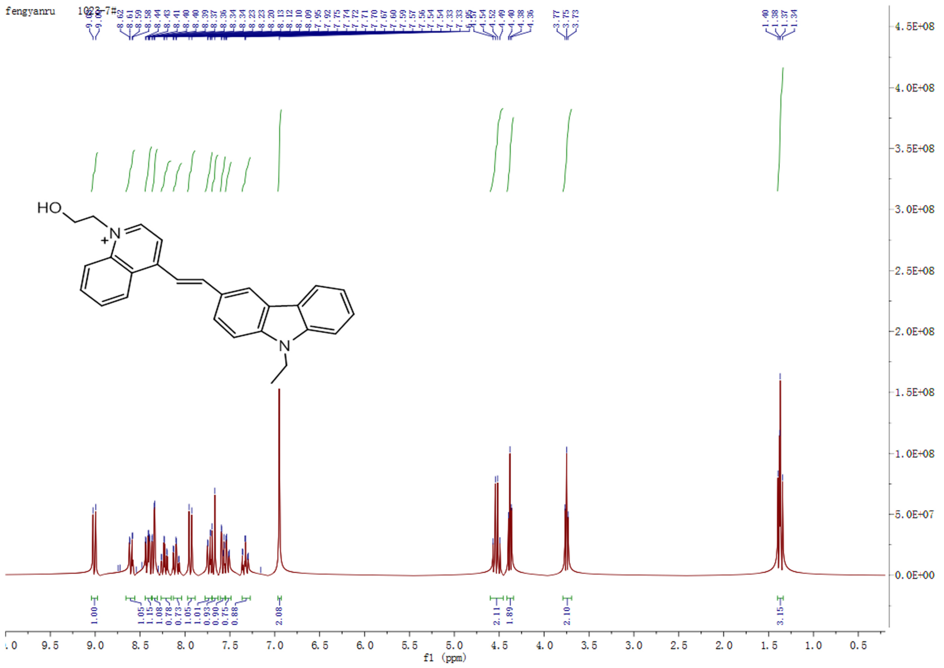

Figure S8 ^{1}H-NMR of H4 (400 MHz, DMSO-d6)

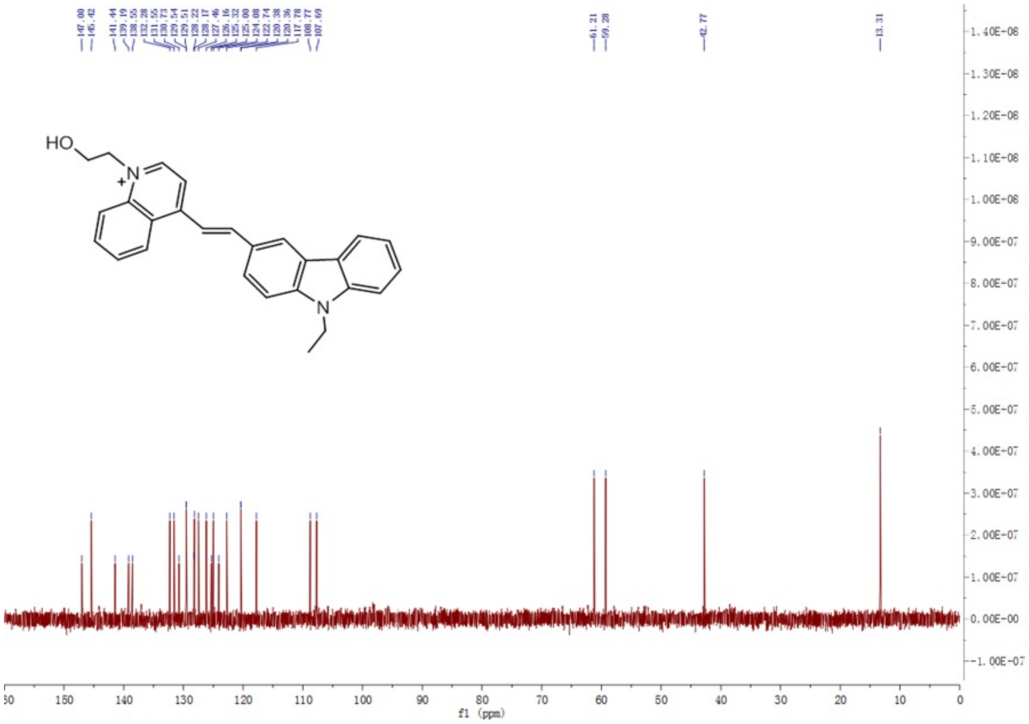

Figure S9 ^{13}C-NMR of H4 (125 MHz, DMSO-d6)

H4:

Mp: 257.2~257.5 °C; ^{1}H-NMR (400 MHz, DMSO-*d6*) δ (ppm): 7.33~9.03 (m, 13H), 6.95 (s, 2H), 4.52 (q, 2H), 4.38 (t, 2H), 3.75 (t, 2H), 1.40 (s, 1H), 1.37 (t, 3H). ^{13}C-NMR (125 MHz, DMSO-*d6*) δ (ppm): 147.00, 145.42, 141.44, 139.19, 138.55, 132.28, 131.55, 130.73, 129.54, 129.51, 128.22, 128.17, 127.46, 126.16, 125.32, 125.00, 124.08, 122.74, 120.38, 120.36, 117.78, 108.77, 107.69, 80.99, 61.21, 59.28, 42.77, 13.31. MALDI-TOF-MS: m/z calculated for $[C_{27}H_{25}N_2O]^+$, 393.50; experiment: 393.268.

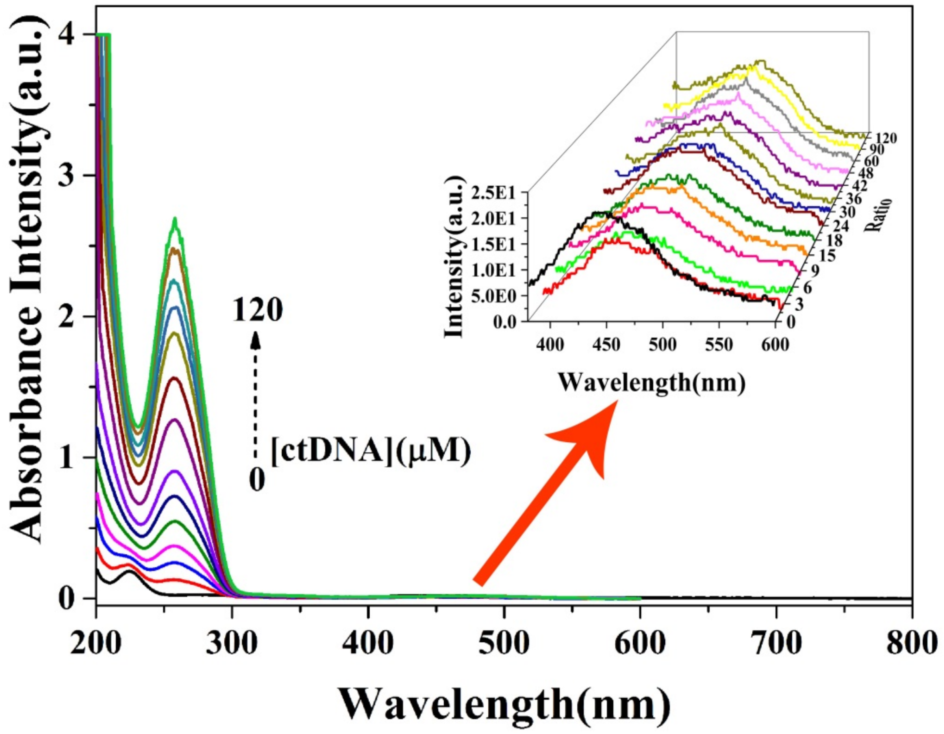

Figure S10 UV-Vis spectrum of H2 according [ctDNA] increased

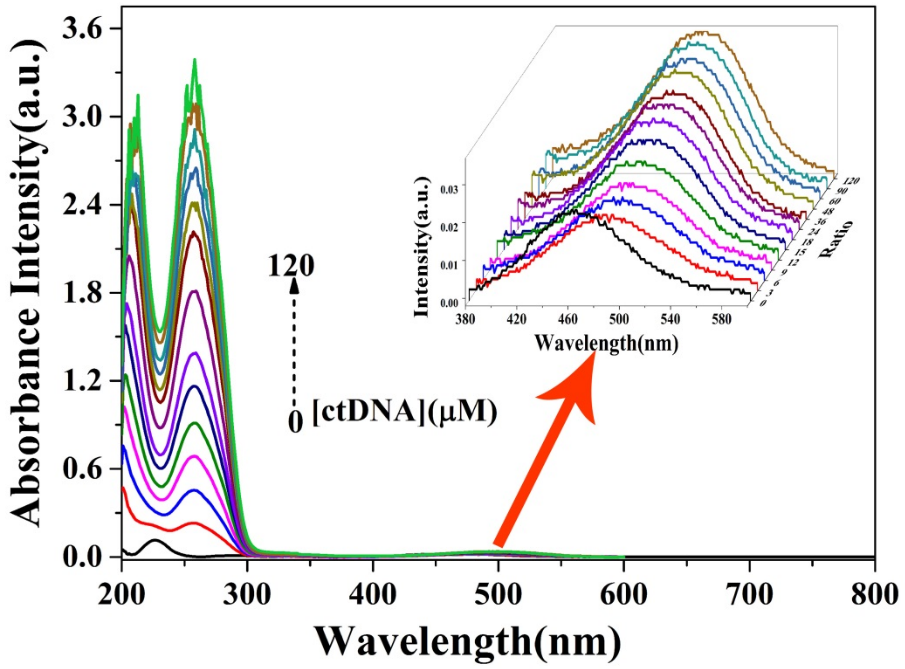

Figure S11 UV-Vis spectrum of M4 according [ctDNA] increased

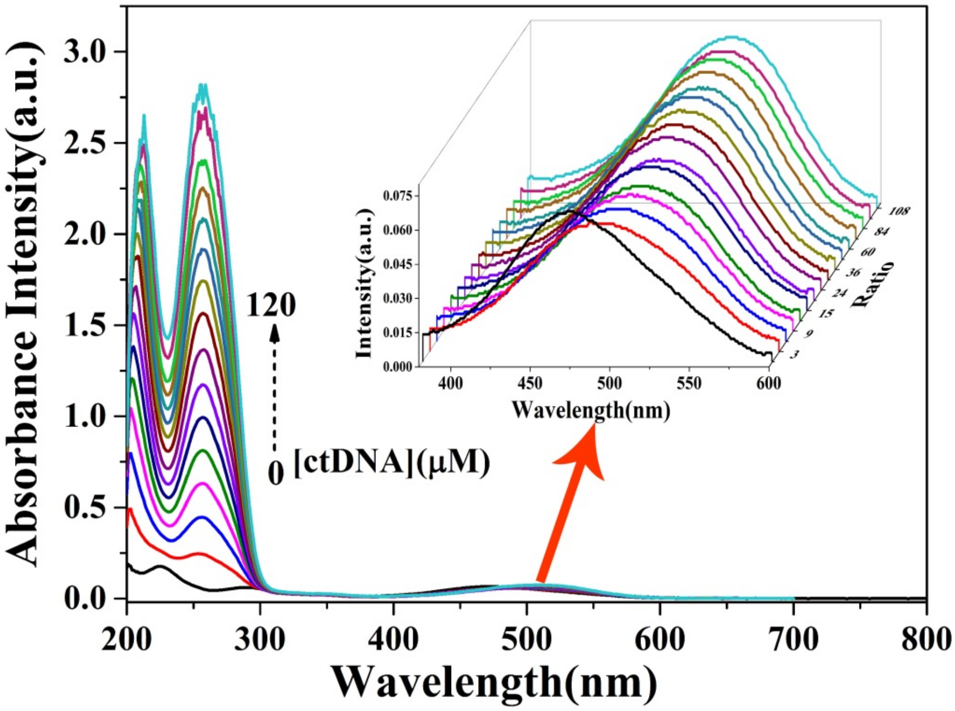

Figure S12 UV-Vis spectrum of H4 according [ctDNA] increased

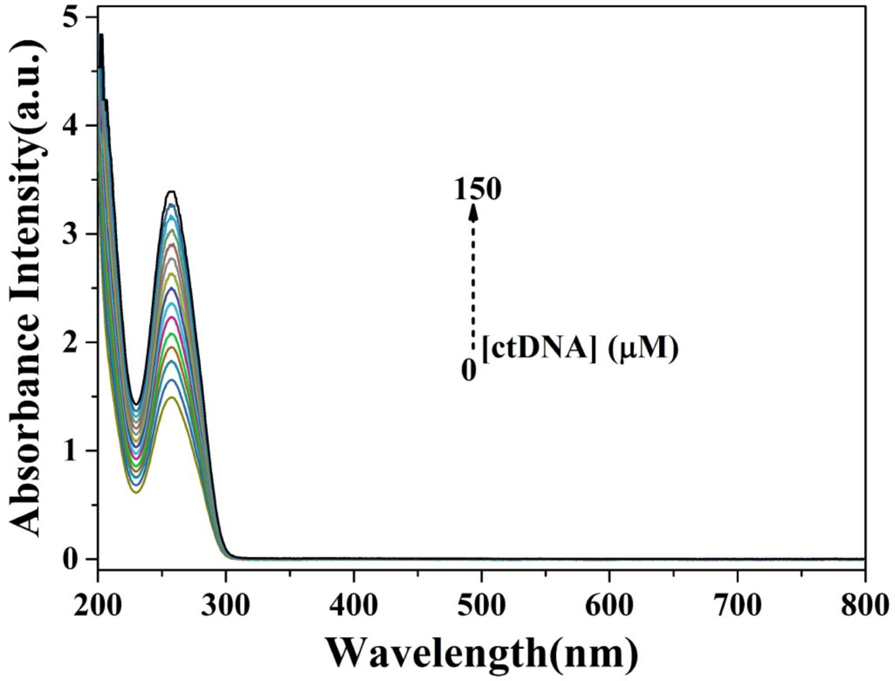

Figure S13 UV-Vis spectrum of blank Tris-HCl buffer solution (pH=7.20) according [ctDNA] increased

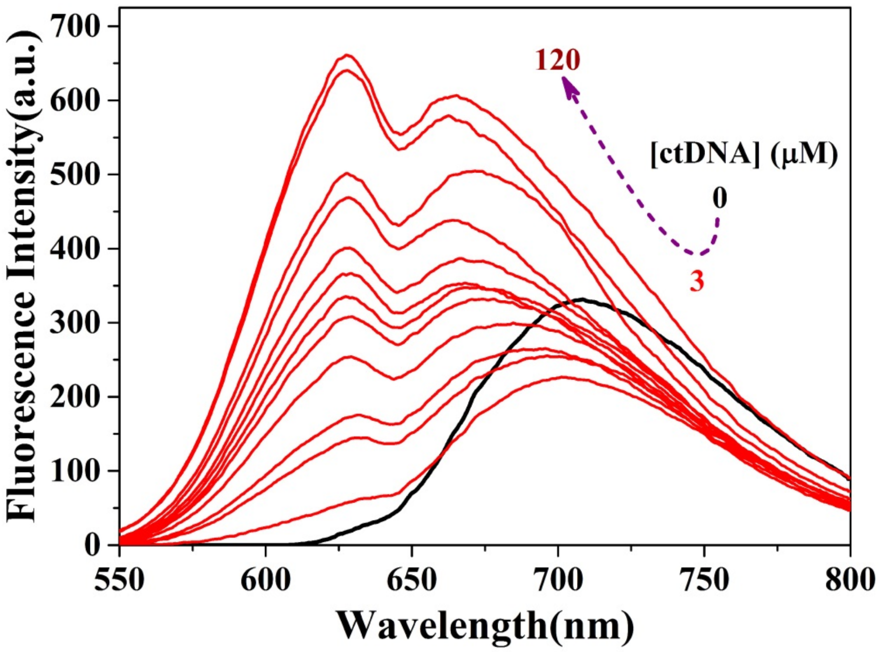

Figure S14 Normalized fluorescence spectrum of H2 according [ctDNA] increased

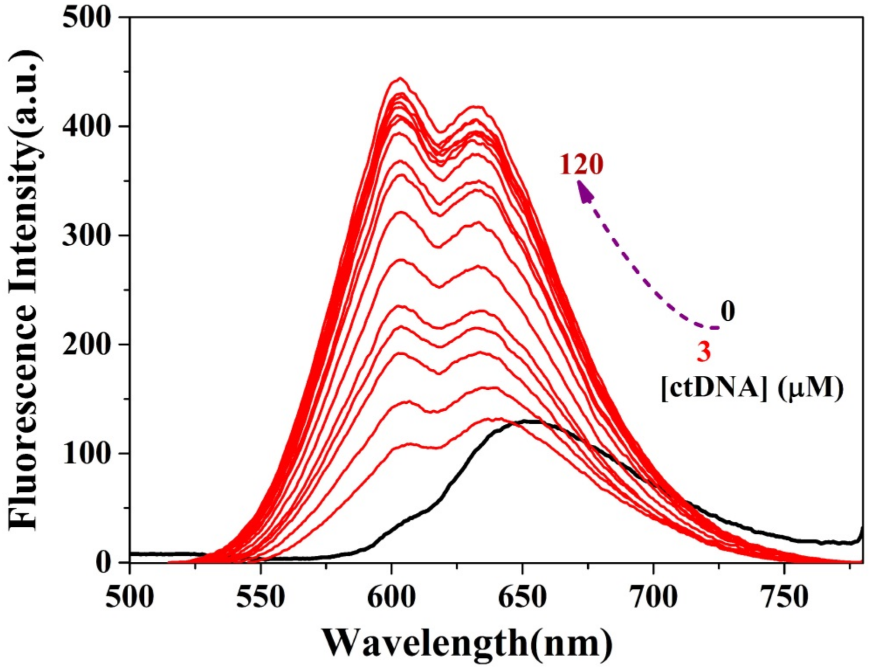

Figure S15 Normalized fluorescence spectrum of M4 according [ctDNA] increased

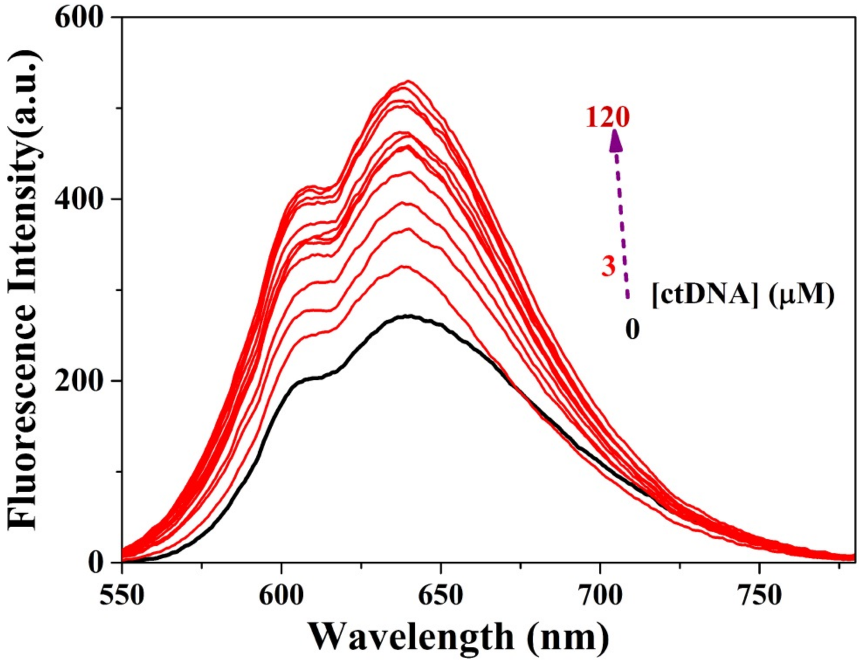

Figure S16 Normalized fluorescence spectrum of H4 according [ctDNA] increased

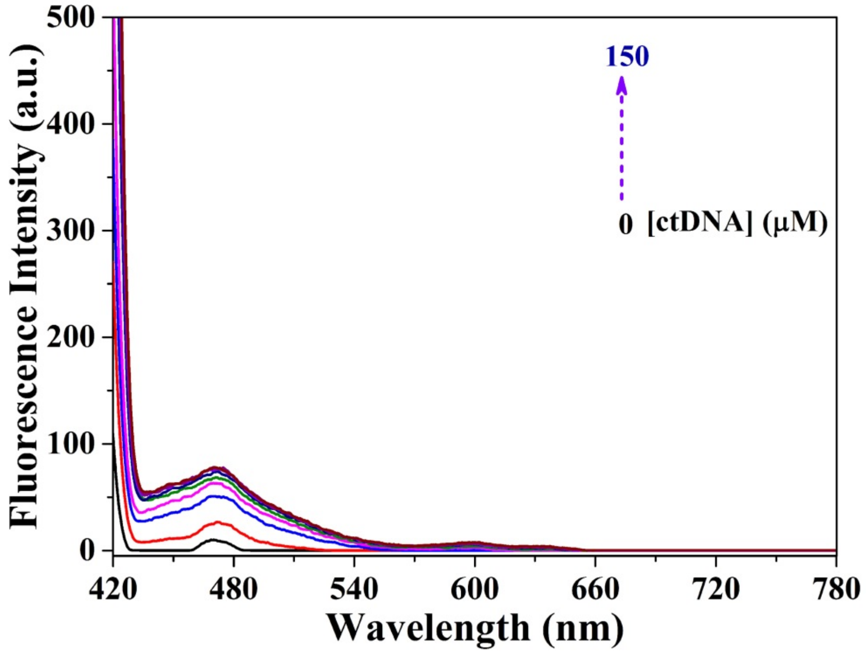

Figure S17 Normalized fluorescence spectrum of blank Tris-HCl buffer solution (pH=7.20) according [ctDNA] increased

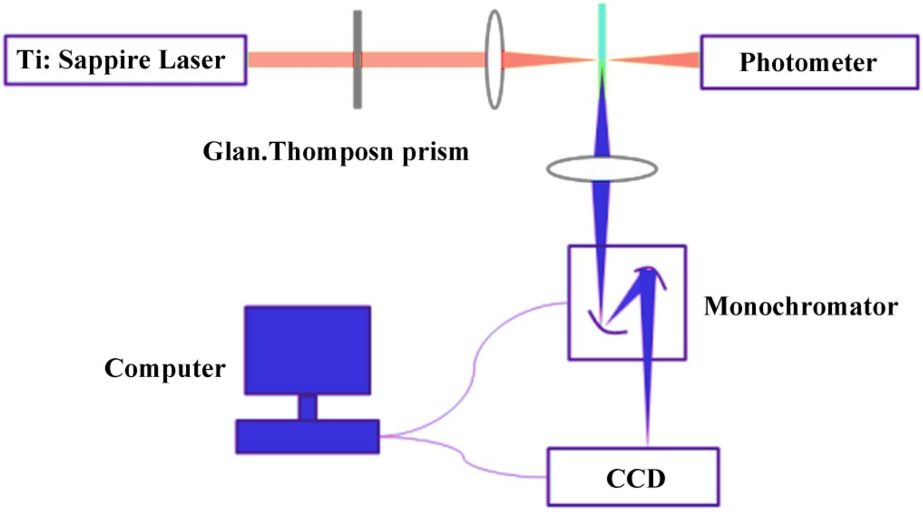

Fig. S18 Experiment apparatus schematic diagram for two-photon-excited properties

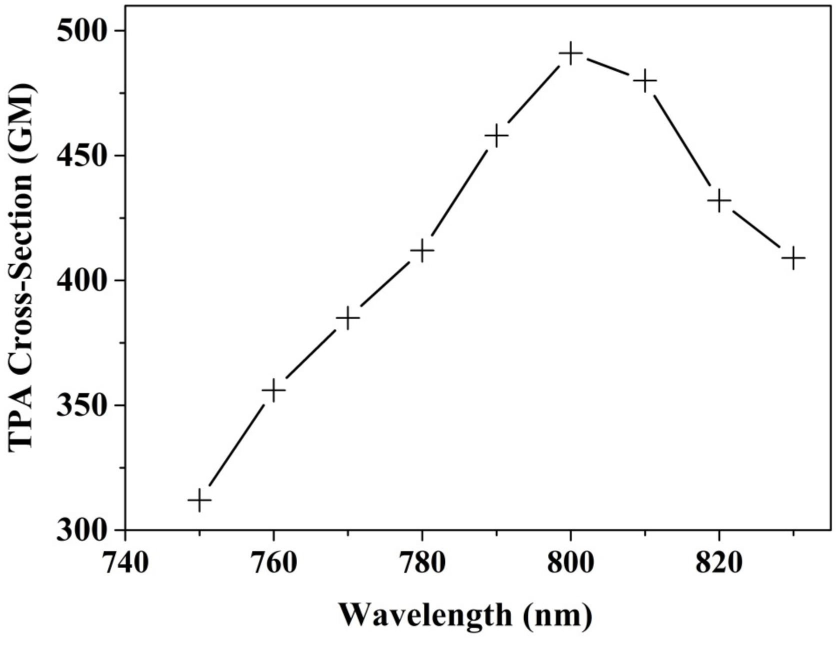

Figure S19 TPA cross-sections of M4 under different incident wavelength

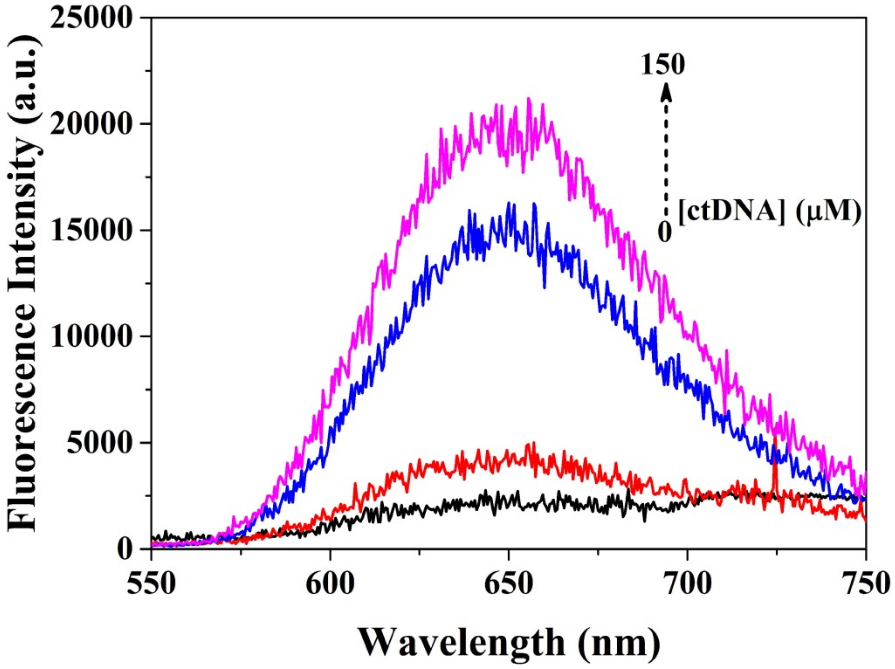

Figure S20 Two-photon excited fluorescence spectrum of H2 according [ctDNA] increased

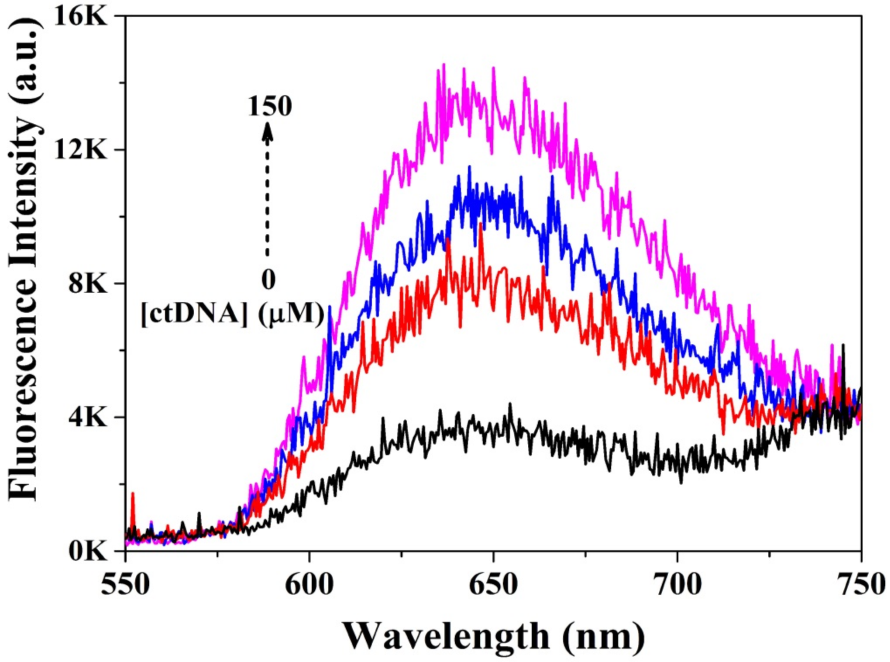

Figure S21 Two-photon excited fluorescence spectrum of M4 according [ctDNA] increased

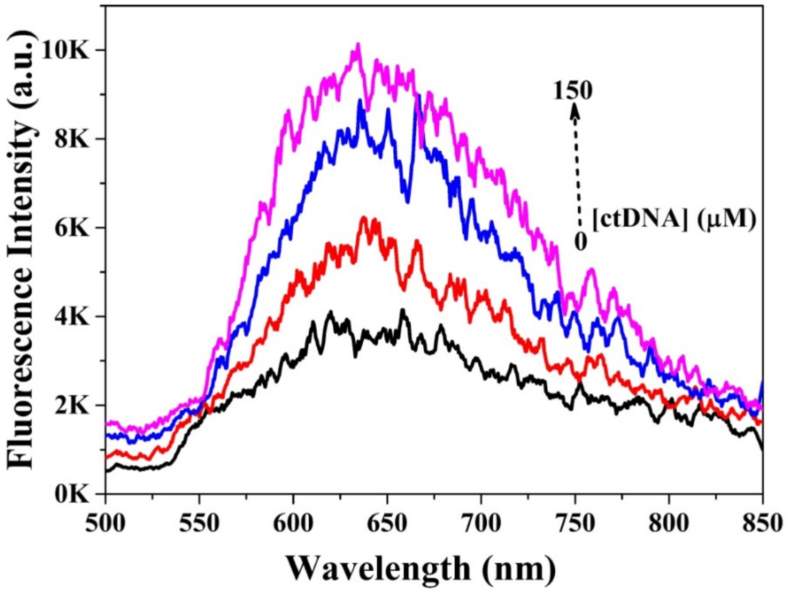

Figure S22 Two-photon excited fluorescence spectrum of H4 according [ctDNA] increased

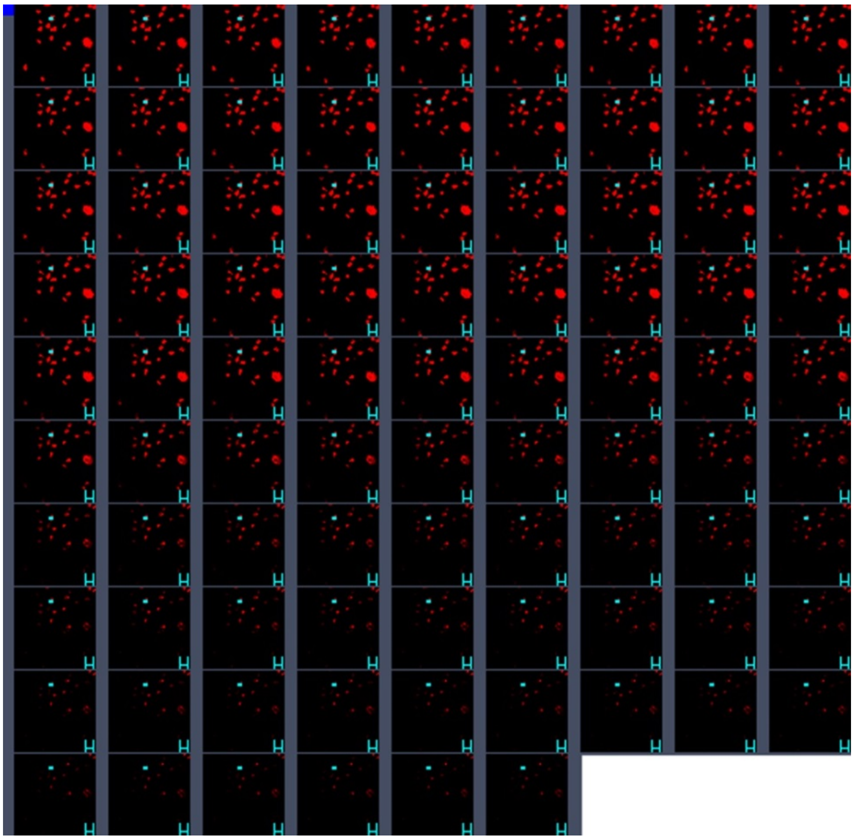

Figure S23 Time-dependent two-photon confocal fluorescence images of 3T3 cell nucleus stained with H2 (0.3 μM) for 15 min, λ_{ex} =800nm, λ_{em} = 600-650 nm. Scale bar was 20 μm. Photograph interval 60 seconds.

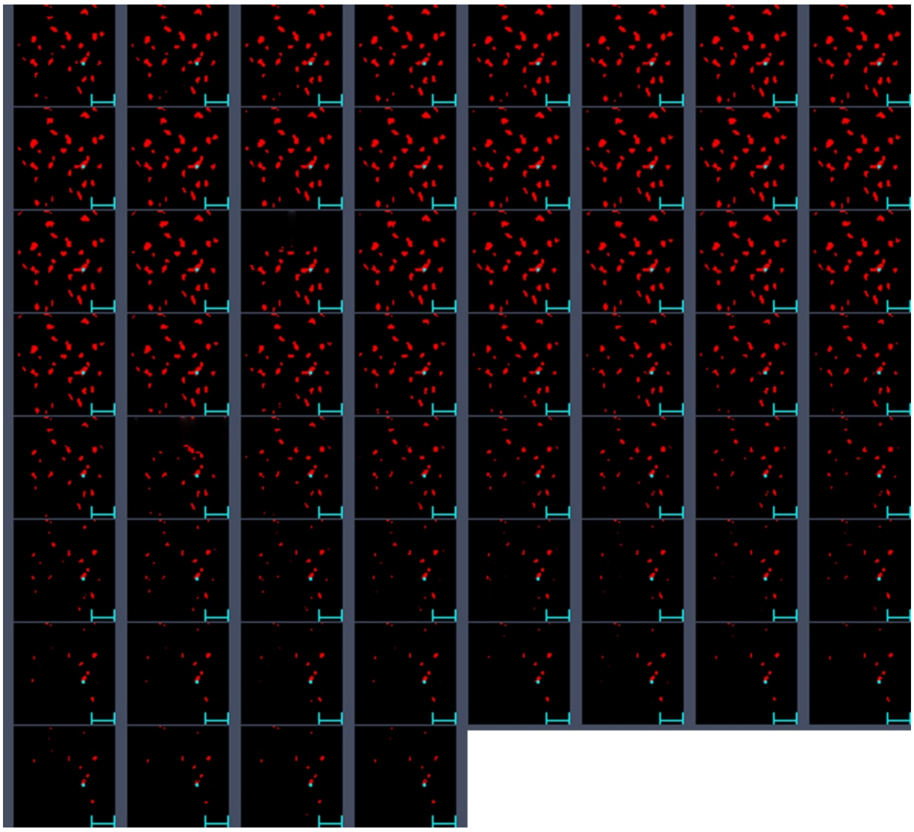

Figure S24 Time-dependent two-photon confocal fluorescence images of 3T3 cell nucleus stained with M4 (0.3 μM) for 15 min, λ_{ex} =800nm, λ_{em} = 600-650 nm. Scale bar was 20 μm. Photograph interval 60 seconds.

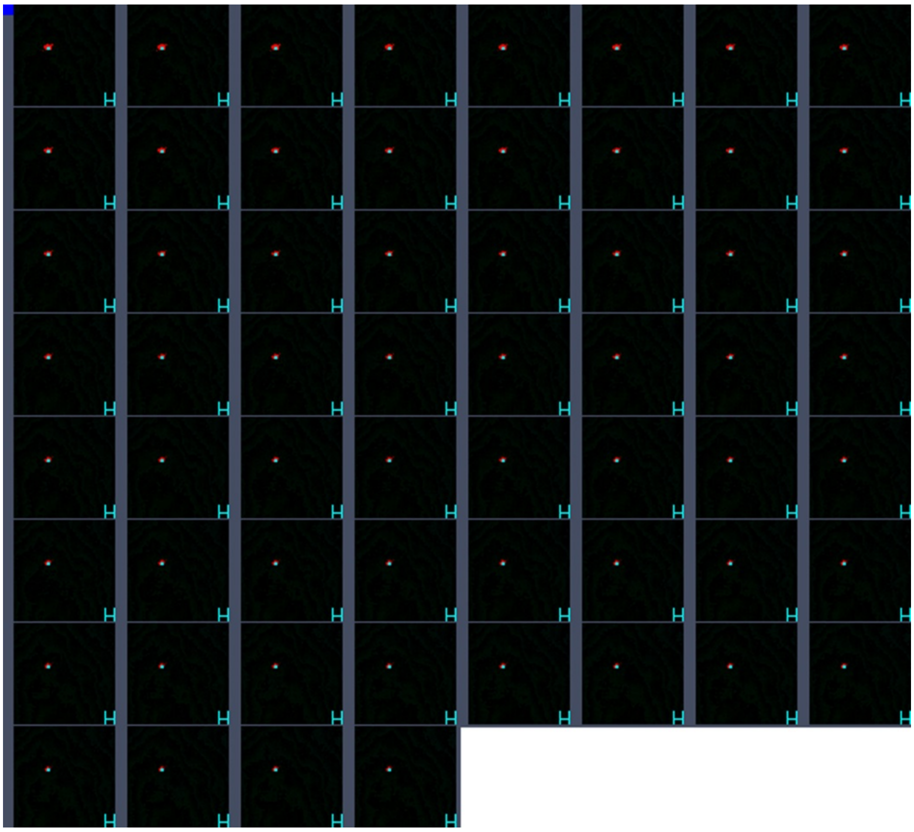

Figure S25 Time-dependent two-photon confocal fluorescence images of 3T3 cell nucleus stained with H4 (0.3 µM) for 15 min, λ_{ex} =800nm, λ_{em} = 600-650 nm. Scale bar was 20 µm. Photograph interval 60 seconds.

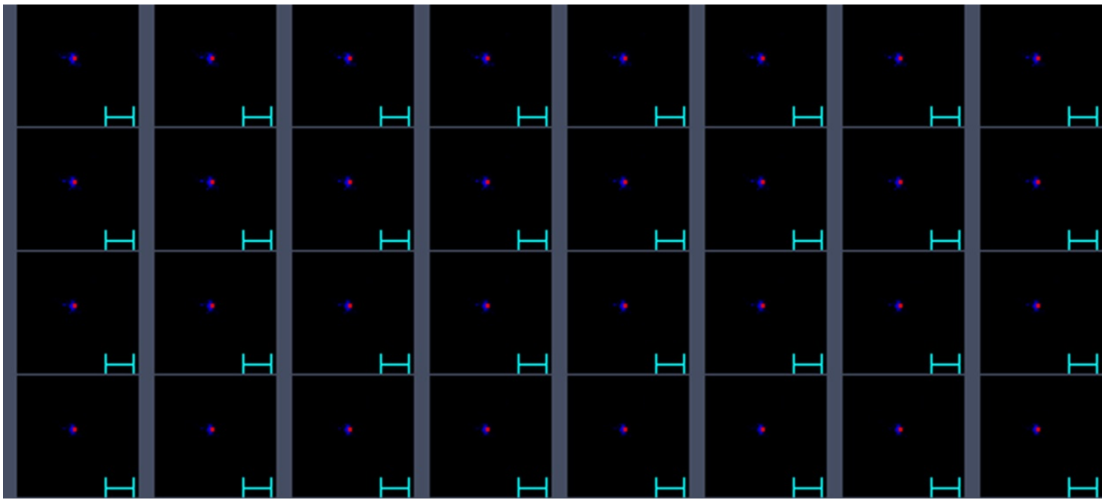

Figure S26 Time-dependent two-photon confocal fluorescence images of 3T3 cell nucleus stained with DAPI (0.3 µM) for 15 min, λ_{ex} =740nm, λ_{em} = 410-490 nm. Scale bar was 20 µm. Photograph interval 60 seconds.
